# Quasi-asymmetric response of the Indian summer monsoon rainfall to opposite phases of the IOD

**DOI:** 10.1038/s41598-017-18396-6

**Published:** 2018-01-09

**Authors:** Swadhin K. Behera, J. V. Ratnam

**Affiliations:** Application Laboratory, Japan Agency for Marine-Earth Science and Technology, Yokohama, Japan

## Abstract

The El Niño/Southern Oscillation has been traditionally linked to the extremes in the Indian summer monsoon rainfall (ISMR) affecting more than a billion people in the region. This trans-oceanic influence is seen to be moderated by the Indian Ocean Dipole (IOD) phenomenon in recent decades. In the presence of a positive IOD (pIOD), the otherwise subdued ISMR in an El Niño year remains close to normal even in the face of record breaking El Niños. While this general influence of pIOD on ISMR is understood, the influence of negative IOD (nIOD) on ISMR is not yet recognized. In this study, it is revealed that those opposite phases of IOD are associated with distinct regional asymmetries in rainfall anomalies. The pIOD is associated with a tripolar pattern in rainfall anomalies with above normal rainfall in central parts of India and below normal rainfall to north and south of it. Conversely, the nIOD is associated with a zonal dipole having above (below) normal rainfall on the western (eastern) half of the country. This spatial quasi-asymmetry arises from the differences in the atmospheric responses and the associated differences in moisture transports to the region during contrasting phases of the IOD.

## Introduction

The summer monsoon variability over the Indian subcontinent is extensively studied owing to its strong impacts on the socio-economic conditions involving a large human population in the region. Past studies have elucidated El Niño/Southern Oscillation (ENSO) as the major driver of monsoon variability. In fact, the Southern Oscillation was discovered while investigating the major Indian famines of the late 19^th^ century^1^. Though the tropical Pacific phenomenon remains as a major aspect in the monsoon variability^2–7^, latter’s associations with other climate variations in the Indian and Atlantic Oceans have emerged in recent years. One of them is the Indian Ocean Dipole (IOD)^8–10^. The frequent emergence of the IOD is said to have weakened the otherwise robust relationship between ENSO and monsoon^11–16^.

The IOD phenomenon modulates the meridional circulation in the region by inducing anomalous convergence patterns over the Bay of Bengal and strengthening of the monsoon trough over central India as seen in the typical IOD year of 1994^11^. This relationship between IOD and Indian summer monsoon rainfall (ISMR) was firmly established^12,13^ with long time-series of observational data and atmospheric model experiments. It is found that during pIOD years, such as that in 1997, when the ENSO co-occurred with the positive phase of the IOD, the ENSO-induced anomalous subsidence is neutralized by the anomalous IOD-induced convergence over the Bay of Bengal^12,13^. This explains why India recorded a near-normal seasonal rainfall during the summer of 1997 despite the presence of a record breaking El Niño in that year.

The IOD normally evolves in late spring, matures in early fall and decays thereafter^8,9^. However, some inter-event variations are noticed in initiation and termination phases of the phenomenon; some of IODs develop early and decay early (early IOD) while some other evolve early but decay late (prolonged IOD). In spite of these minor variations, the IOD and the monsoon have a common phase of evolution/occurrence during June-September. Comparing the variability in the phases of their evolutions, a recent study suggested that an early IOD also plays a significant role, like normal and prolonged IOD, in enhancing ISMR even though the strengths of those IODs are generally weaker compared to other IODs^16^. The excess evaporation from the Arabian Sea together with the stronger cross-equatorial flow leads to the enhanced monsoon activity in those early pIOD years^16^. Hence, the IOD link to ISMR is generally stable irrespective of the period of initiation and the lifetime of those events.

While these previous studies have shown in general the influence of IOD on the ISMR, in the light of diminishing ENSO impacts, the regional asymmetries, or to that mater the symmetries, arising from IOD teleconnections to India are neither studied nor discussed. Here we report for the first time the existence of symmetries and the asymmetries in ISMR responses to opposite phases of the IOD.

## Results

**Figure 1:**
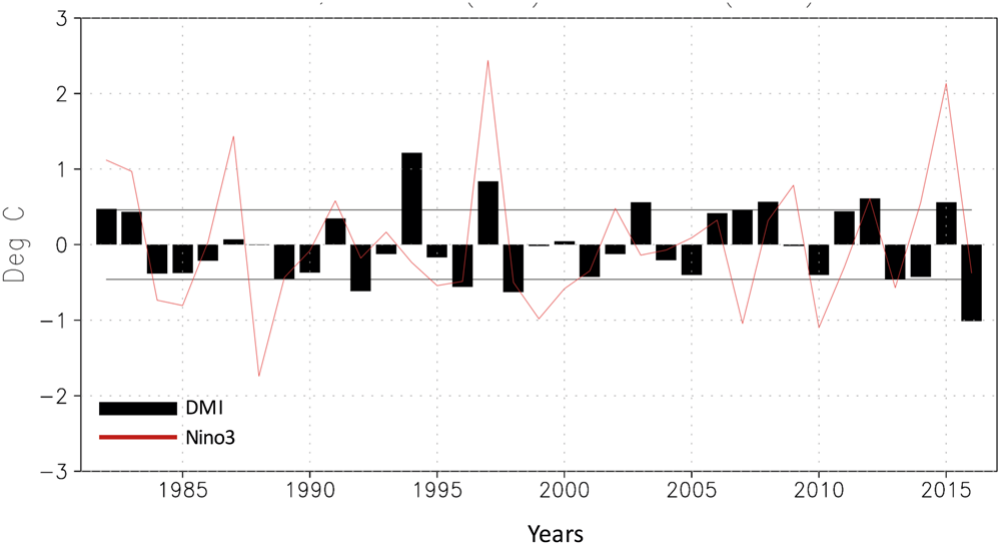
June-September averaged anomalies of DMI and Niño3. The anomalies are derived from the OISST anomalies as described in the Methods section. The horizontal lines represent ±1 standard deviation.

**Figure 2:**
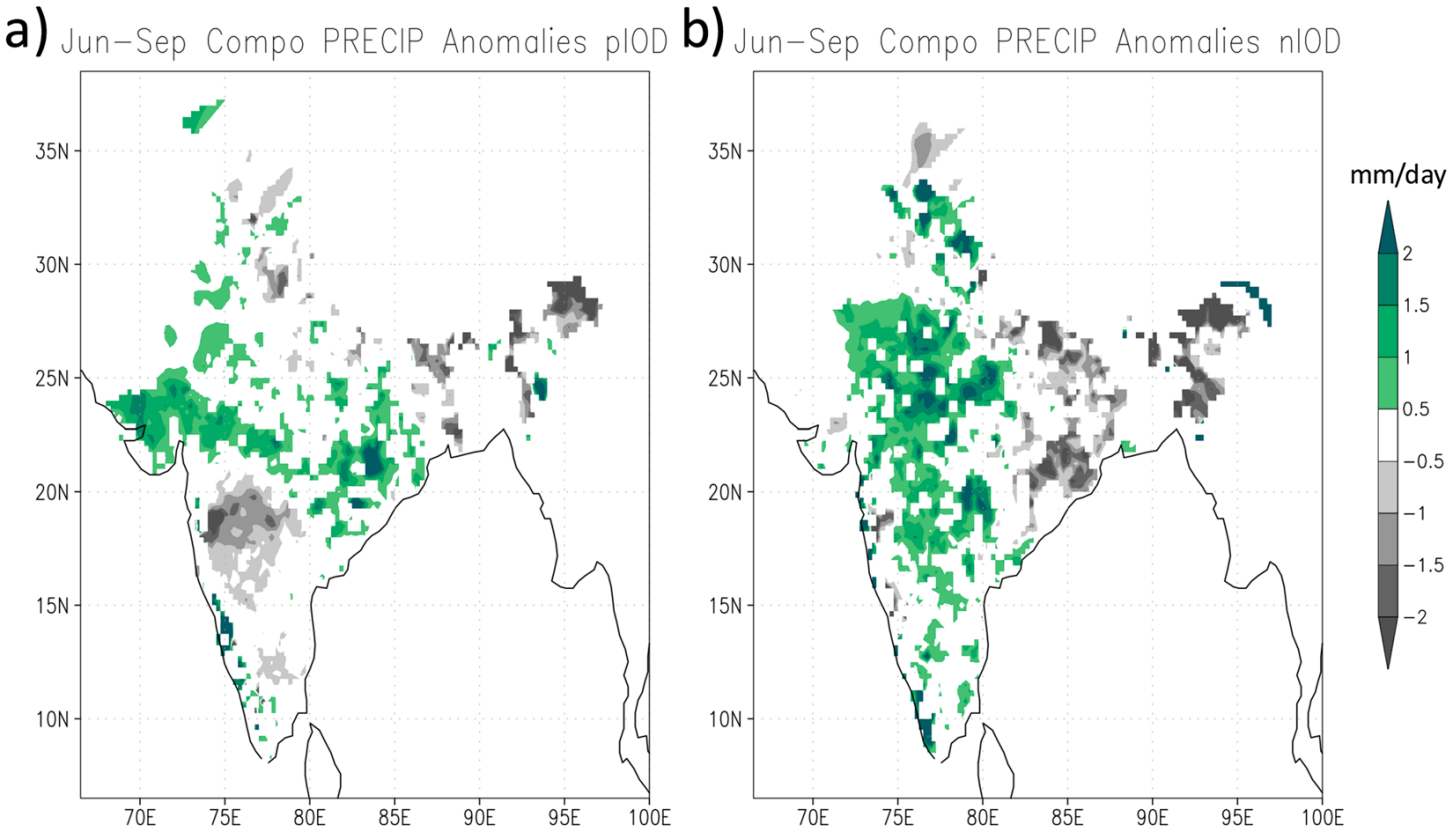
June-September composite of rainfall anomalies for (**a**) pIOD and (**b**) nIOD events based on IMD daily rainfall data. Shown values exceed 90% of confidence level using a 2-tailed t-test. This figure is plotted using GNU Public License software GrADS.

The ISMR variability related to opposite phases of the IOD is investigated by selecting pIOD and negative IOD (nIOD) events based on dipole mode index (DMI) of June-September (Fig. 1). Eight pIOD and five nIOD events were identified since 1982 as explained in the Methods section. The 8 pIOD years are 1982, 1994, 1997, 2003, 2007, 2008, 2012, 2015 and the 5 nIOD years are 1992, 1996, 1998, 2013, 2016, respectively. These years were then used to make composites of different ocean and atmospheric fields. Composite rainfall anomalies, derived from India Meteorological Department (IMD) data, for those events show distinct patterns of symmetric and asymmetric responses to opposite phases of the IOD (Fig. 2a,b). First of all, positive rainfall anomalies are seen over several parts of the country during both phases of the IOD. This is counter intuitive, as one would expect large-scale rainfall deficiencies in response to nIOD (Fig. 2b), in a symmetric sense, since previous studies have documented a positive rainfall response arising from the pIOD. Hence, it is surprising to note that the rainfall response to nIOD, the opposite phase of the pIOD, is not exactly symmetric.

### The asymmetry

The rainfall anomalies in response to pIOD vary meridionally across the subcontinent with positive rainfall anomalies in the middle (covering most parts of central India stretching from western to eastern states) and negative anomalies on both north and south sides of it. In contrast to this meridional tripolar pattern during pIOD, the corresponding anomalies in case of nIOD events are more zonally oriented varying from west to east and represented as a dipolar pattern with positive (negative) anomalies in the central and western (eastern) parts of the country. This gives rise to the regional quasi-asymmetry in the rainfall response to opposite phases of the IOD. It is noted here that the above normal rainfall in the western part of India in the latter case is meridionally covering a wider region from south to north as compared to that during pIODs.

The spatial asymmetries seen in the association between ISMR and both phases of IOD give rise to some interesting regional responses. As a consequence of the commonality in the anomaly patterns associated with both phases, a couple of meteorological subdivisions in the central parts of the country have also asymmetric responses with positive rainfall anomalies during both phases of IOD. On a similar note, the northeastern states suffer from negative anomalies having on the drier sides during both phases of IOD.

**Figure 3:**
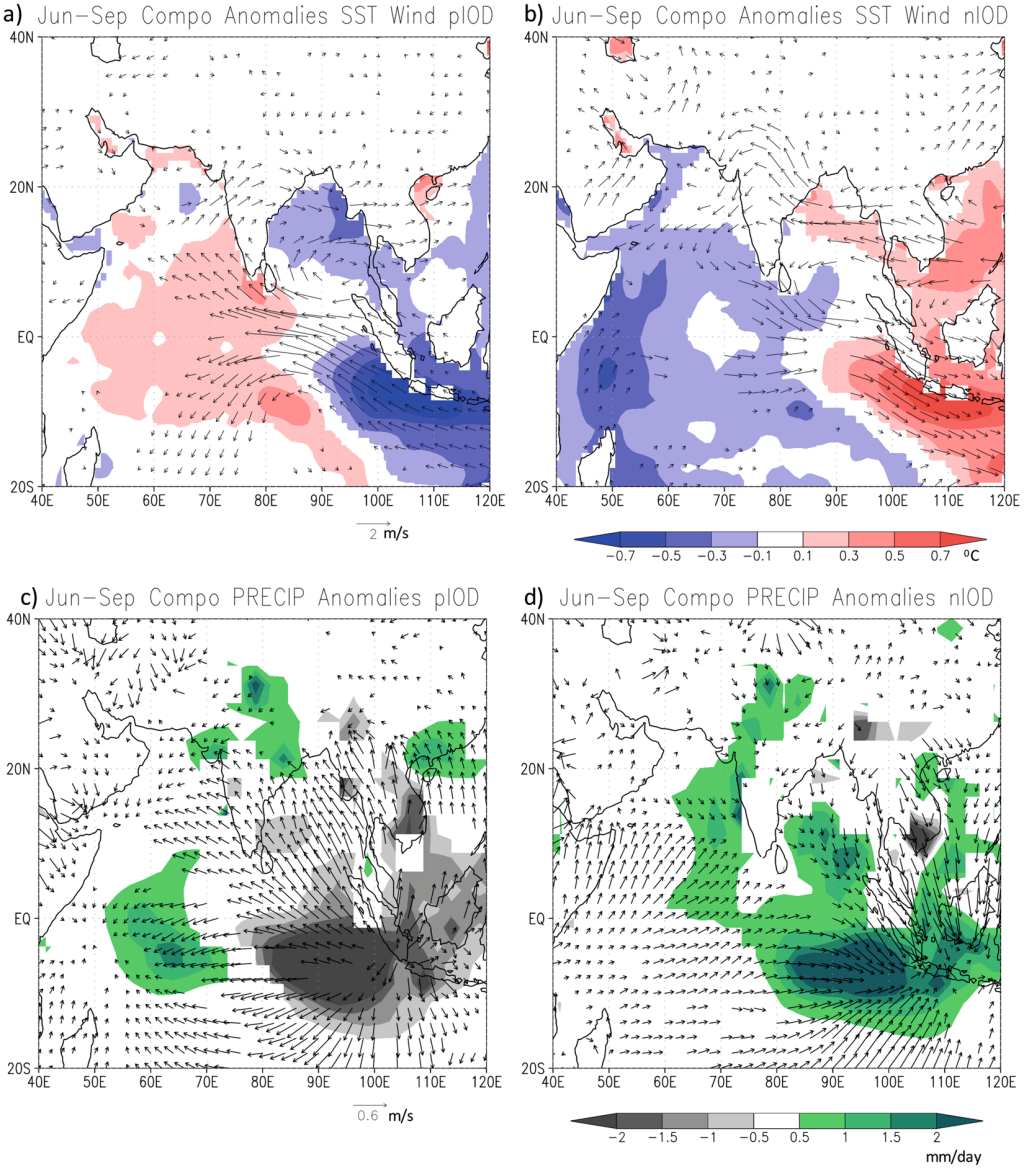
June-September composite anomalies of SST (shaded) and 850 hPa wind for (**a**) pIOD and (**b**) nIOD. The corresponding anomalies for rainfall and 850hPa divergent wind are shown in (**c**) and (**d**). Shown values exceed 90% of confidence level using a 2-tailed t-test. This figure is plotted using GNU Public License software GrADS.

In order to understand the mechanisms of such asymmetric responses, we analyze here the large-scale SST, rainfall and other variabilities associated with both phases of IOD. The composites of SST and wind anomalies show clear evolutions of pIOD and nIOD during the season (Fig. 3a,b). The cold (warm) anomalies near the Sumatra coast in pIOD (nIOD) composite is accompanied by anomalous easterlies (westerlies) and divergent (convergent) winds in the lower troposphere of the eastern Indian Ocean (Fig. 3c,d). Associated with these surface conditions, we find suppressed (enhanced) rainfall anomalies in the eastern Indian Ocean in the pIOD (nIOD) composite (Fig. 3c,d).

The composited rainfall anomalies over the Indian sub-continent derived from the Global Precipitation Climatology Project (GPCP) rainfall analyses (Fig. 3) match well with those of the IMD rainfall analysis (Fig. 2). As in the composites of IMD rainfall data, asymmetric responses are seen in the composites of the GPCP rainfall anomalies though with minor variations. Some parts of the sub-continent selectively do (or do not) get anomalous rainfall during both phases of the IOD. The meridional tripole and zonal dipole are clearly seen to be associated with pIOD and nIOD events, respectively. This contrasting feature is diagnosed through the composites of the 850hPa divergent wind anomalies. The composite anomalies show a clear zonal contrast with convergence on the western side of India during nIOD events (Fig. 3c,d).

### Mechanism

**Figure 4:**
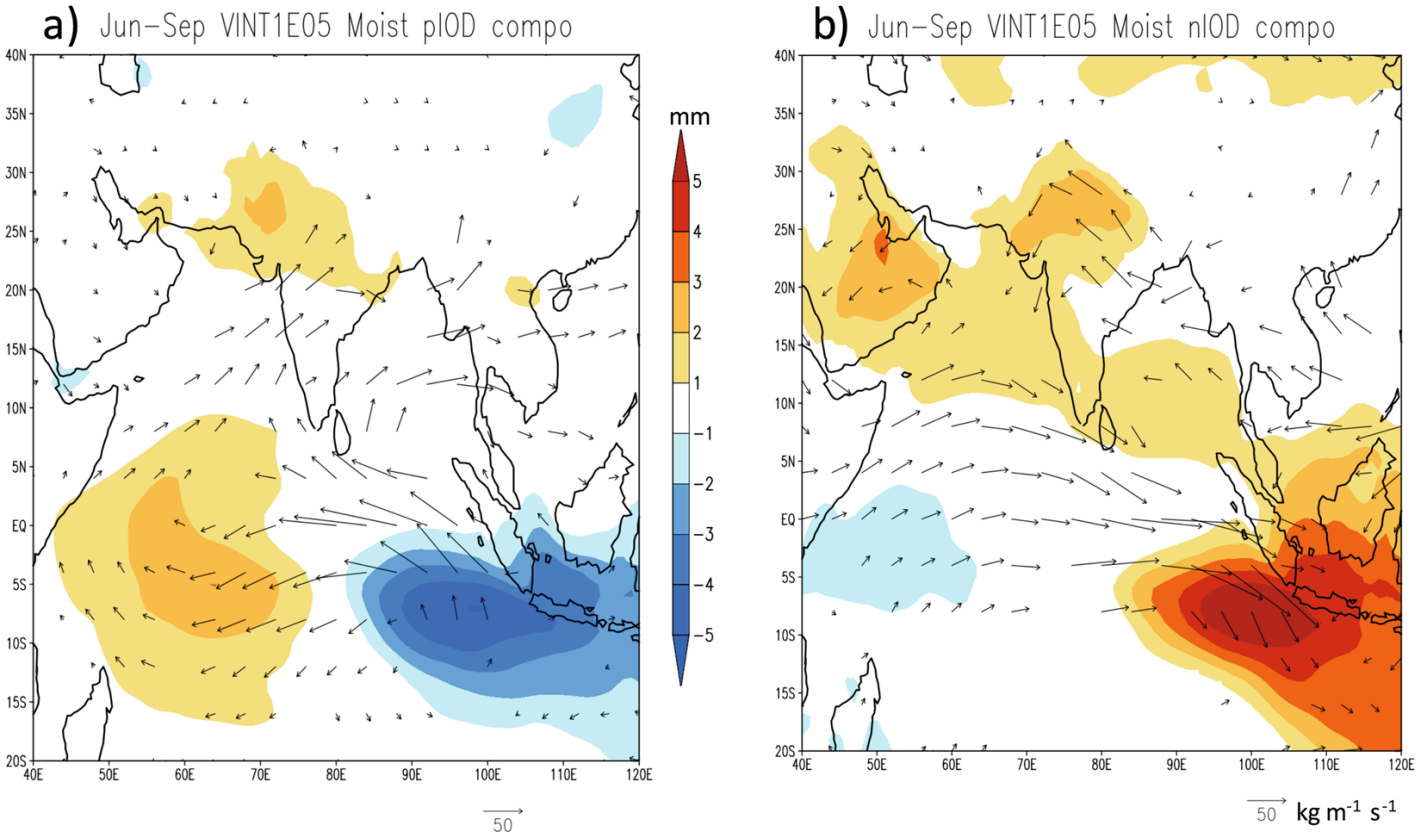
June-September composites of vertically integrated (surface to 300 hPa) moisture transport anomalies (vector) and specific humidity anomalies (shaded) for (**a**) pIOD and (**b**) nIOD events. Shown values exceed 90% of confidence level using a 2-tailed t-test. This figure is plotted using GNU Public License software GrADS.

The interesting difference in the Indian rainfall response to both phases of IOD is explained by the corresponding differences in moisture transports as well as in zonal and meridional monsoon circulations. The composites of height-integrated tropospheric moisture transports show a clear difference in the way moisture is transported to the Indian sub-continent during opposite phases of the IOD. The asymmetric response to both phases of IOD is clearly seen in tropospheric moisture (Fig. 4, shaded) and transport vectors. Positive (negative) tropospheric moisture anomalies together with anomalies of easterly (westerly) transports are seen over the eastern Indian Ocean (Fig. 4a,b) during pIOD (nIOD). In fact, the composite plot of tropospheric moisture for pIOD appears similar to what has already been shown earlier in relation to the 1994 event^11^. The diverged moisture anomalies from the eastern Indian Ocean are then transported to the central parts of India. The westerly transport anomalies to the south of 25°N turn to easterly anomalies near the head of the Bay of Bengal. This is in a sense surface intensification of the meridional monsoon circulation and characteristic strengthening of the monsoon trough normally observed in the monsoon season, leading to moisture accumulation (yellow shadings in Fig. 4a) and abundant rainfall there (Fig. 2a). At the same time, the northward intensification of the transport vectors (Fig. 4a) gives rise to the moisture divergence in the southern parts of the country.

The moisture transport over the Indian sub-continent associated with nIOD is quite asymmetric to that of pIOD (Fig. 4b), as is the condition in the equatorial regions. Associated with a lower-level convergence in the eastern Indian Ocean, which pulls the air mass toward Sumatra, the westerly anomalies are confined to equatorial regions – thereby missing the Indian sub-continent. This situation favors easterly anomalies north of 15°N at 850 hPa (Fig. 3b) as well as in tropospheric moisture transports (Fig. 4b). As a result, there is a strong moisture divergence (convergence) in the eastern (western) parts of India and hence accumulation of moisture in the western parts of the country (Fig. 4b) that favors abundant rainfall there (Fig. 2b).

**Figure 5:**
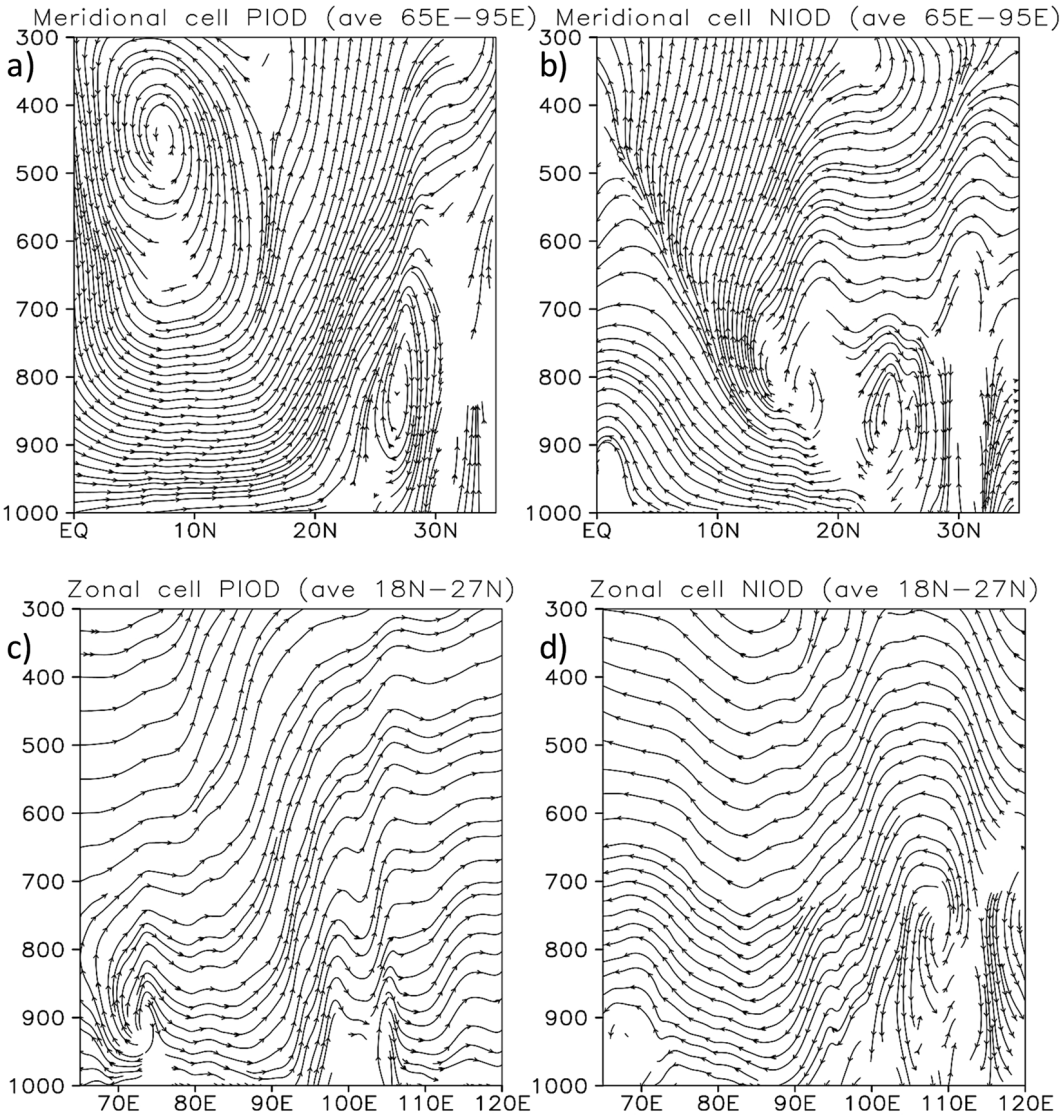
June-September composites of anomalous meridional circulation cells averaged between 65°E and 95°E (**a**) for pIOD and (**b**) for nIOD events. The corresponding anomalous zonal circulation cells averaged between 18°N and 27°N are shown in (**c**) and (**d**), respectively. This figure is plotted using GNU Public License software GrADS.

The zonal and meridional asymmetries in rainfall is further diagnosed by using the composites of meridional and zonal circulation cells over the Indian sub-continent. The meridional circulation cells averaged between 65°E and 95°E show interesting differences between pIOD and nIOD composites (Fig. 5a,b). The pIOD composite shows a clear meridional cell with rising air (Fig. 5a) around the monsoon trough corresponding to anomalously higher moisture (Fig. 4a) and rainfall (Fig. 2a) there. On the other hand, the meridional cell in the nIOD case is rather disrupted indicating lesser role of the meridional monsoon circulation in those nIOD years. Interestingly a zonal circulation cell averaged between 18°N and 27°N shows subsidence on the eastern parts of the country (Fig. 5d) supporting the negative moisture (Fig. 4b) and rainfall (Fig. 2b) anomalies there. This anomalous zonal cell is formed by ascending air on the eastern side in the South China Sea. That region gets anomalously higher rainfall during the nIOD events (Fig. 3d) and hence the rising air from that region spread eastward to subside over the Bay of Bengal and eastern parts of India. On the other hand, the western parts of the country are not affected by this descending branch and hence the rainfall is above normal on that side. Such a zonal variation is not apparent in the composite plot of pIOD (Fig. 5c). Uniform rising air throughout the monsoon trough region (18°N-27°N) supports the rainfall distribution for the pIOD case. The role of different SST and other boundary conditions need further investigation using numerical models. But with known biases in coupled general circulation models^17^, perhaps sensitivity to different boundary conditions needs further studies with regional models^18,19^.

### In relation to ENSO

**Figure 6:**
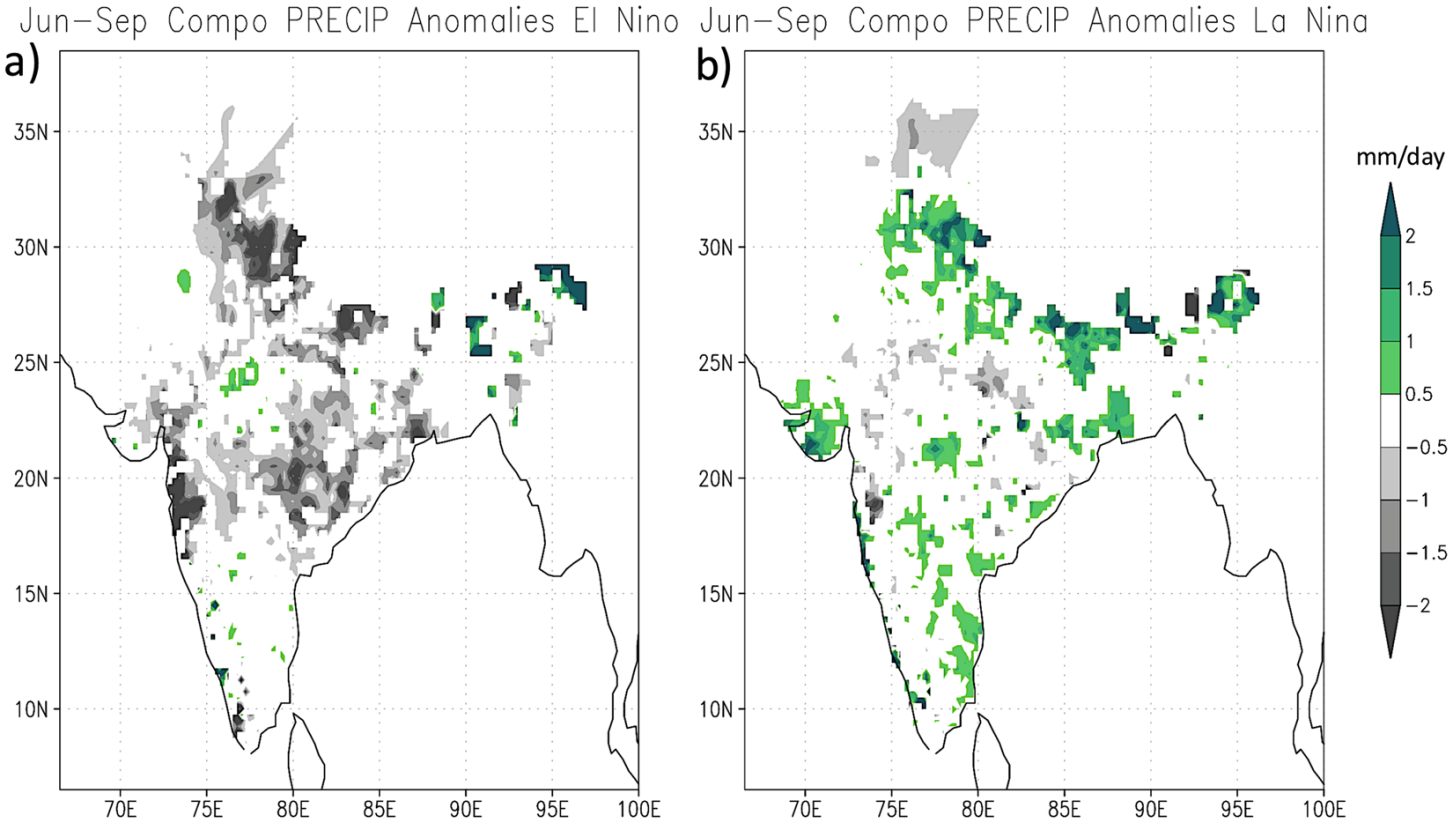
June-September composite of rainfall anomalies for (**a**) El Niño and (**b**) La Niña events based on IMD daily rainfall data. Shown values exceed 90% of confidence level using a 2-tailed t-test. This figure is plotted using GNU Public License software GrADS.

The concurrence of some of the IOD and ENSO events is often discussed in terms of their dependence on each other as well as on their teleconnections. Therefore, we have investigated the differences in the responses arising from El Niño/La Niña compared to pIOD/nIOD. We will be comparing pIOD with El Niño, and nIOD with La Niña because those combinations are favored by in-phase Walker Circulations of both oceans when both phenomena are interlinked. Figure 6 shows the composites of the rainfall anomalies over India during El Niño and La Niña years. The years used for the El Niño composite are 1982, 1983, 1987, 1997, 2009 and 2015 and the corresponding years for the La Niña composite are 1984, 1985, 1988, 1999, 2007 and 2010. These years are picked when the amplitude of Niño3 index (shown in Fig. 1) was close to 1 standard deviation. Comparing these years with the corresponding years of pIOD and nIOD (discussed earlier), we notice that none of 6 La Niña years correspond to nIODs. 3 out of 6 El Niño events (1982, 1997 and 2015) co-occurred with pIOD events. This suggests a possible interlink during some of the pIOD years that actually reduces the effect of El Niño on ISMR: The 4 other pIODs that evolved independent of El Niño and La Niña occurrence clearly suggest the pIOD link to the anomalously higher rainfall over the central parts of the country as explained earlier. Moreover, it is found that one of the pIOD events co-occurred with a La Niña (2007) not supporting the in-phase Walker circulations of both basins. Considering the heterogeneity in their occurrences it is difficult to clearly connect their variations. In any case, the differences in their associations with ISMR are very clear when we compared the rainfall composites of El Niño with that of pIOD (Figs 6a and 2a) and La Niña with that of nIOD (Figs 6b and 2b).

The composite rainfall anomalies show a clear symmetric response between El Niño and La Niña. The composite for El Niño shows negative rainfall anomalies spanning all over the country except for a few patches over the Northeast (Fig. 6a). On the other hand, the La Niña composite shows a wetter than normal condition in general all over the country (Fig. 6b) except for a few states in the central parts: It may be noted that these are the states that receive more than normal rainfall during the pIODs. This is quite a contrast since the responses related to pIOD could neither be opposite nor similar to that of La Niña. In addition, it is noted that the rainfall anomalies in the western half of the country are higher during nIOD events as compared to La Niña events. Therefore, although there are anticipated variations in rainfall characteristics during the years when ENSO and IOD co-occur, the unrelated pIOD and nIOD years show a distinct asymmetric response (Figs. 2,3,4) as against a symmetric response in rainfall to both phases of ENSO (Fig. 6).

## Discussions

The impact of ENSO on the summer monsoon rainfall over India has been discussed for several decades^2–4^ following the seminal work of Walker^1^. This link is in fact a matter of discussions and debate even today. With the availability of longer time-series and better quality observational data together with the simulation results from state of the art numerical models, we have better insights now on the processes associated with ENSO, monsoon and other climate variations. A common understanding at this stage is that the El Niño (La Niña), the warm (cold) phase of ENSO, is linked to below (above) normal rainfall in most parts of India. This is to some extent corroborated in the composite plots of Fig. 6 though both phases are not entirely affecting the rainfall over the whole country.

In the recent couple of decades, however, we have come across another mode of climate variations in the Indian Ocean, the Indian Ocean Dipole. It is demonstrated that the IOD has an overarching impact on the ISMR variability in recent decades^12,13^. Those inferences are even extended to pre-observational period based on coral records in Kenya and Sumatra^20,21^. Particularly the Kenya coral records revealed apparent complementary interdecadal changes of ENSO–ISMR and Indian Ocean Dipole (IOD)–ISMR relationships^21^. This shift in the relationships is attributed to the decadal shift in the occurrence of pIOD, which has become more frequent in recent decades^22^. While these studies, and some others cited in the first part of this article, have brought out the overall IOD relationship with the ISMR, characteristic responses and spatiotemporal variability in ISMR responses to opposite phases of IOD are not discussed and hence the associated mechanisms are not recognized. This is what motivated us to study the differences in the responses of both phases of IOD and their associations with ISMR.

## Conclusion

It is for the first time here we have shown that the ISMR response may not necessarily be spatially coherent to both phases of IOD as one would logically conclude based on the responses observed in opposite phases of ENSO. The central-western part (75°E–80°E and 20°N–25°N) of India, the region of seasonal monsoon trough, has a symmetric response with above normal rainfall in both phases of the IOD. However, we see asymmetric responses south and east of the monsoon trough region (Figs. 2 and 3). These symmetric and asymmetric responses arise due to the nature of teleconnection and moisture distributions over India during pIOD and nIOD. The moisture transports during pIOD strengthen the monsoon trough and the meridional-monsoon circulation (Fig. 4). This gives rise to abundant rainfall around the monsoon trough through an intensified monsoon-Hadley circulation but below normal rainfall south and north of the trough forming a distinct meridional tripolar pattern in the rainfall anomalies.

The situation is different in nIOD case when the atmospheric responses and the moisture distribution favor moisture divergence in the eastern part but moisture convergence in the western part of the country. In addition, a zonal circulation cell is seen with anomalously rising air over South China Sea and subsidence over eastern parts of India. Those give rise to abundant rainfall on the western part and forms a zonal dipole with the drier eastern part. In addition to revealing the asymmetric nature, here we have discussed the mechanisms underlying that asymmetrical behaviors in the ISMR. The ensued regional asymmetry is a unique feature associated with the ISMR response to IOD and is not recognized well in model predictions. We hope our observational findings will help to develop an effective seasonal prediction system for both phases of the IOD.

## Methods

### Material

Meteorological data such as wind and divergent wind anomalies are derived from NCEP/NCAR reanalysis^23^. Anomalies of vertically integrated specific humidity and moisture transports are derived from ERA-interim reanalysis^24^. Anomalies of specific humidity were vertically integrated from surface to 300hPa and the anomalies were derived from a climatology for the base period of 1982 to 2010. The precipitation anomalies are derived from GPCP monthly precipitation dataset based on global station data and gridded on 2.5° × 2.5° degrees. In addition to GPCP rainfall, we have used high-resolution daily rainfall data of IMD^25^. The IMD data is at a resolution of 0.25° × 0.25°. The interannual anomalies for these monthly climate fields are derived by removing monthly climatologies from their monthly values. The gridded SST anomalies are derived from OISST^26^. With the availability of satellite data, the SST and other analyses products have become reliable since 1982. Therefore, most of the analyses used here are based on the data since that year.

### Indices

The Niño3 is the domain averaged SST anomalies for the box 150°W to 90°W and 5°N and 5°S. The DMI is the difference in SST anomalies between the western (50°E to 70°E and 10°S to 10°N) and eastern (90°E to 110°E and 10°S to the equator) boxes^8^. IOD years are picked from the DMI index when the yearly averaged values of June-September seasons exceed one standard deviations.

Analyses were done using GNU Public License software GrADS.

## References

[1] Walker, G.T. Correlations in seasonal variations of weather. IX. Mem. India Meteorological Department (1924).

[2] Rasmusson EM, Carpenter TH (1983). Relationship between eastern equatorial Pacific sea surface temperatures and rainfall over India and Sri Lanka. Mon Weather Rev.

[3] Webster PJ, Yang S (1992). Monsoon and ENSO: Selectively interactive systems. Q. J. R. Meteorol. Soc..

[4] Kripalani RH, Kulkarni A (1997). Climatic impact of El Niño/La Niña on the Indian monsoon: A new perspective. Weather.

[5] Kumar K, Krishna B, Rajagopalan M, Hoerling G, Bates M (2006). Cane,: Unraveling the Mystery of Indian Monsoon Failure During El Niño. Science.

[6] Rajeevan M, Pai DS (2007). On the El Niño-Indian monsoon predictive relationships. Geophys. Res. Lett..

[7] Azad S, Rajeevan M (2016). Possible shift in the ENSO-Indian monsoon rainfall relationship under future global warming. Sci. Rep..

[8] Saji NH, Goswami BN, Vinayachandran PN, Yamagata T (1999). A dipole mode in the tropical Indian Ocean. Nature.

[9] Yamagata, T. *et al*. Coupled Ocean-Atmosphere Variability in the Tropical Indian Ocean. AGU Book Ocean-Atmosphere Interaction and Climate Variability, C. Wang, S.-P. Xie and J.A. Carton (eds.), Geophys. Monogr., 147, AGU, Washington D.C., 189–212 (2004).

[10] Webster PJ, Moore A, Loschnigg J, Leban M (1999). Coupled dynamics in the Indian Ocean during 1997–1998. Nature.

[11] Behera SK, Krishnan R, Yamagata T (1999). Unusual ocean–atmosphere conditions in the tropical Indian Ocean during 1994. Geophys. Res. Lett..

[12] Ashok K, Guan Z, Yamagata T (2001). Impact of the Indian Ocean Dipole on the relationship between the Indian Monsoon rainfall and ENSO. Geophys. Res. Lett..

[13] Ashok K, Guan Z, Saji NH, Yamagata T (2004). Individual and Combined Influences of ENSO and the Indian Ocean Dipole on the Indian Summer Monsoon. Geophys. Res. Lett..

[14] Loschnigg J, Meehl GA, Webster PJ, Arblaster JM, Compo GP (2003). The Asian Monsoon, the Tropospheric Biennial Oscillation, and the Indian Ocean Zonal Mode in the NCAR CSM. J. Clim..

[15] Gadgil, S., Vinayachandran, P.N., Francis, P.A., Gadgil, S., Extremes of the Indian summer monsoon rainfall: ENSO and equatorial Indian Ocean Oscillation. Geoph. Res. Lett. 31 (L12213), 10.1029/2004GLO19733, (2004).

[16] Anil, N., M.R. Ramesh Kumar, R. Sajeev and P.K. Saji, Role of distinct flavours of IOD events on Indian summer monsoon. Natural Hazards, 10.1007/s11069-016-2245-9 (2016).

[17] Pradhan M (2017). Shift in MONSOON–SST teleconnections in the tropical Indian Ocean and ENSEMBLES climate models’ fidelity in its simulation. Int. J. Climatol..

[18] Bhaskar Rao DV, Ashok K, Yamagata T (2004). A Numerical Simulation Study of the Indian Summer Monsoon of 1994 using NCAR MM5. J. Meteor. Soc. Japan.

[19] Ratnam JV, Behera SK, Krishnan R, Doi T, Ratna SB (2017). Sensitivity of Indian summer monsoon simulation to physical parameterization schemes in the WRF model, Clim. Res..

[20] Abram NJ, Gagan MK, Cole JE, Hantro WS, Mudelsee M (2008). Recent intensification of tropical climate variability in the Indian Ocean. Nat. Geosci..

[21] Nakamura N (2009). Mode shift in the Indian Ocean climate under global warming. Geophys. Res. Lett..

[22] Cai W, Cowan T, Sullivan A (2009). Recent unprecedented skewness towards positive Indian Ocean dipole occurrences and its impact on Australian rainfall. Geophys. Res. Lett..

[23] Kanamitsu et al, Bulletin of the American Meteorological Society,1631–1643 (2002).

[24] Dee DP (2011). The ERA-Interim reanalysis: configuration and performance of the data assimilation system. Q.J.R. Meteorol. Soc..

[25] Pai DS (2014). Development of a new high spatial resolution long period daily gridded rainfall data set over Indian and comparison with existing data over the region. Mausam.

[26] Reynolds (2002). An improved *in situ* and satellite SST analysis for climate. J. Climate.

